# Multi-Modal GAN-Based Anomaly Detection for Signal Reliability Assessment in Fabric-Integrated Smart Textiles

**DOI:** 10.3390/s26165158

**Published:** 2026-08-14

**Authors:** Jianbin Wu, Ru Fan, Xiangfang Ren

**Affiliations:** 1 School of Design, Jiangnan University, 1800 Lihu Avenue, Wuxi 214122, China; jerrywu@jiangnan.edu.cn; 2 School of Automation and Intelligent Science, Jiangnan University, Wuxi 214122, China; 6251928001@stu.jiangnan.edu.cn

**Keywords:** smart textiles, fabric-integrated sensors, signal reliability, multi-modal anomaly detection, generative adversarial network

## Abstract

Smart textiles require reliable physiological sensing despite signal degradation caused by fabric deformation, material fatigue, and unstable textile–skin interfaces. This study presents a multi-modal GAN-based anomaly detection framework for signal reliability assessment using acceleration (ACC), electrodermal activity (EDA), and heart rate (HR). Controlled injection of baseline drift, amplitude scaling, and signal dropout generates normal/anomalous labels for supervised training. Temporal encoding and cross-modal attention distinguish textile-mimicking anomalies from physiologically plausible variations. On the 36-subject PhysioNet dataset, the framework achieves an F1-score of 0.9197 and exceeds the evaluated single-modal models by more than 35%. Using PhysioNet-trained weights without fine-tuning, zero-shot evaluation on the textile-integrated WWBS Metrics dataset achieves an F1-score of 0.8723 with ACC and derived HR. These results demonstrate cross-dataset transfer under the controlled synthetic-fault protocol; validation using physically induced textile faults remains necessary.

## 1. Introduction

Smart textiles constitute an important class of industrial textile systems in which sensing, computation, and communication functions are embedded directly into fibrous structures and fabric substrates through textile-compatible engineering and fabrication processes. Rather than serving solely as novel wearable devices, such systems are increasingly deployed in industrial contexts that demand long-term operational stability, including healthcare monitoring (Medtech), performance-oriented sports garments (Sporttech), and occupational safety and protective equipment (Protech) [1,2,3,4]. Compared with conventional rigid wearables, textile-integrated systems offer superior conformability, breathability, and mechanical compliance, enabling continuous physiological monitoring under prolonged wear and dynamic motion conditions [5].

From an industrial textile engineering perspective, the primary value of smart textiles extends beyond enhanced wearability to their role as distributed, fabric-integrated sensing systems operating throughout the full garment lifecycle. During real-world use, such systems are continuously subjected to repeated mechanical deformation, long-term daily wear, and varying environmental conditions, all of which directly affect signal acquisition and transmission. This deep system-level integration of sensing functions within textile substrates fundamentally differentiates smart textiles from conventional electronics-mounted wearables, in which sensors are mechanically isolated from the supporting structure. As a consequence, smart textile systems face unique engineering challenges related to signal integrity, interfacial stability, and long-term reliability that must be explicitly addressed to enable scalable industrial deployment rather than laboratory-level demonstrations [6].

However, the practical deployment of smart textiles in industrial applications is still constrained by persistent reliability challenges that directly affect system viability and product lifetime. As highlighted in prior studies, maintaining washability and long-term durability remains a fundamental bottleneck for e-textiles, since repeated laundering can damage electronic components, alter conductive pathways, and progressively degrade sensor performance, posing a critical challenge for large-scale manufacturing and quality assurance [7,8]. Beyond laundering-related degradation, smart textile systems are continuously exposed to textile-specific stressors during daily wear. These include substrate fatigue, cyclic mechanical deformation [9], and unstable interfaces between conductive fibers and electronic circuits. Intermittent transmission interruptions induced by fabric bending or creasing are also common [10]. Several recent studies have highlighted that reliability and long-term deployability remain key bottlenecks preventing smart textile technologies from large-scale industrial adoption [11,12,13]. These factors can directly compromise the integrity and consistency of acquired physiological signals—such as acceleration (ACC), electrodermal activity (EDA), and heart rate (HR)—leading to distorted signal patterns and potentially erroneous analytical outcomes if left undetected [14].

Consequently, despite advances in textile materials [15] and flexible energy solutions for wearable systems [16], material- or device-level improvements alone remain insufficient to ensure reliable long-term operation under real-world textile conditions. In practical deployments, smart textile systems must continuously operate in the presence of deformation-induced disturbances, intermittent sensor degradation, and partial modality failures, for which offline signal correction or post hoc data cleaning is neither timely nor adequate.

This limitation highlights the need for an embedded, system-level intelligent paradigm that operates directly at the point of data acquisition. Such a paradigm must continuously assess signal reliability, perform online anomaly diagnosis, and, critically, distinguish system-level faults from physiologically plausible extreme states. These faults typically originate from textile-integrated sensors and interconnects. Establishing such a capability is a prerequisite for enhancing the reliability of smart textile systems and meeting performance, safety, and quality requirements for industrial deployment.

In this context, advances in artificial intelligence provide an enabling tool for addressing reliability engineering challenges in smart textile systems [17]. Beyond improving data interpretation or personalized feedback [18], extending intelligent methods toward embedded [19] assessment of data quality—namely, enabling online signal reliability monitoring in textile systems—represents a critical step toward building reliable and trustworthy next-generation smart textiles. Recent studies on multi-modal smart textiles for artificial sensation and physiological monitoring further demonstrate the potential of fusing heterogeneous sensory information to enhance system-level robustness and performance [20,21]. Nevertheless, much of the existing research remains focused on sensing material development [22] or high-level back-end data analytics, lacking systematic modeling and online detection strategies tailored to the multi-source, heterogeneous signal failure modes inherent to textile-integrated systems. This gap substantially limits the transition of smart textile technologies from laboratory prototypes to industrial-grade, high-reliability products.

Several AI-based approaches have been explored to enhance sensor reliability in wearable and textile-integrated systems. Existing methods include anomaly detection using autoencoders or isolation forests, fault classification using support vector machines or random forests, and signal reconstruction using recurrent neural networks or Kalman filters. While these methods have shown promise in rigid wearable applications, they are not directly transferable to smart textiles for two reasons. First, most operate on single-modal signals and do not exploit the complementary information available from multiple sensor modalities such as ACC, EDA, and HR, which is particularly detrimental in textile systems where individual modalities may fail intermittently. Second, existing approaches have not been designed to address textile-specific failure characteristics, including deformation-induced dropout, gradual conductive path degradation, and fabric-bending-related intermittent connectivity, which differ substantially from the failure modes of conventional rigid wearables. Consequently, multi-modal anomaly detection tailored to textile-specific signal degradation remains insufficiently investigated.

GAN-based wearable-data methods provide a relevant generative modeling context for the proposed framework. For example, Kamruzzaman et al. used a Wasserstein GAN to augment wearable heart-rate and activity data before applying a cokurtosis-based anomaly detector [23]. Their framework uses generative augmentation and a downstream statistical detector, whereas the present work uses adversarial representation learning within a supervised multi-modal detector for ACC, EDA, and HR reliability assessment. VAE-based probabilistic fusion has likewise been explored for multi-modal fault tolerance [24]. The present architecture was selected to jointly encode temporal dependencies and cross-modal consistency while learning from predefined anomaly labels; it is not claimed to establish a general advantage over all VAE-based approaches.

To address this gap, this study proposes a supervised multi-modal GAN-based anomaly detection framework for fabric-integrated physiological sensing. The framework formulates signal reliability assessment as binary discrimination between normal windows and windows containing textile-mimicking anomalies. It is designed to identify signal-level degradation associated with contact resistance drift, amplitude variation, and intermittent signal loss while reducing confusion with physiologically plausible variations.

The main contributions of this work can be summarized as follows:

1. A controlled anomaly injection strategy for generating labeled multi-modal windows representing baseline drift, amplitude scaling, and signal dropout in fabric-integrated sensing.
2. A supervised multi-modal GAN-based detection strategy that combines temporal encoding, cross-modal attention, and gated fusion to distinguish signal anomalies from genuine physiological responses.
3. Subject-independent evaluation on PhysioNet, including comparisons with single-modality and controlled architectural variants, together with zero-shot cross-dataset evaluation using PhysioNet-trained weights on the textile-integrated WWBS Metrics dataset without fine-tuning.

**Figure 1:**
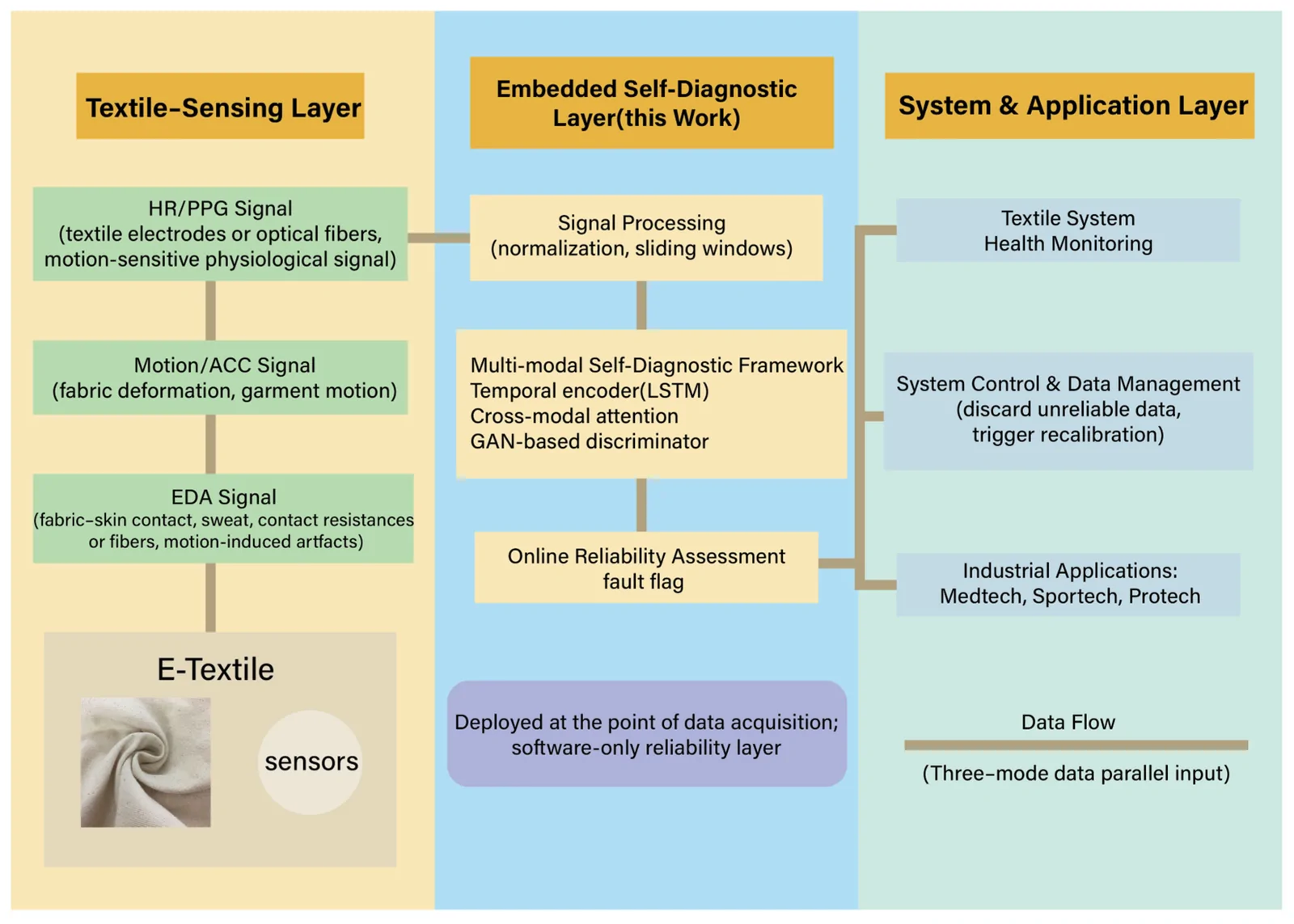
System-level positioning of the proposed embedded anomaly detection framework within an industrial smart textile system. The framework operates as an on-device, software-only reliability layer deployed at the point of data acquisition. It receives multi-modal textile-sensing signals (ACC, EDA, and HR), performs online reliability assessment, and supports system health monitoring, data management, and industrial smart textile applications.

To clarify the industrial positioning of the proposed approach, Figure 1 illustrates the system-level integration of the embedded anomaly detection framework within an industrial smart textile architecture. The framework is intended as an on-device, software-only reliability layer operating between textile-integrated sensing units and downstream communication and application modules. Multi-modal physiological signals acquired from textile-based sensors (ACC, EDA, HR) are first preprocessed and then jointly analyzed to provide real-time assessments of signal reliability. By identifying unreliable or faulty signals before higher-level analytics and application pipelines, the framework is intended to support system health monitoring in Medtech, Sporttech, and Protech applications.

## 2. Materials and Methods

### 2.1. Dataset and Preprocessing

This study uses the public PhysioNet dataset [25,26] for model development and subject-independent evaluation. The dataset was collected with an Empatica E4 wearable and contains blood volume pulse, skin temperature, motion, and electrodermal activity signals. The present analysis uses acceleration (ACC), electrodermal activity (EDA), and heart rate (HR) from 36 participants in aerobic and anaerobic exercise conditions. Signals were normalized and segmented into 30 s windows. The 30 s duration was an engineering choice intended to retain temporal context for the comparatively slow HR and EDA dynamics while maintaining window-level detection latency; it is not presented as a universal standard, and window-length sensitivity remains to be evaluated.

PhysioNet was selected because it provides ACC, EDA, and HR concurrently, includes subject identifiers for subject-independent partitioning, and is publicly available for reproducible model development. However, the Empatica E4 is a rigid wearable, and its recordings cannot be assumed to reproduce the contact mechanics or noise distributions of textile-integrated sensors. Accordingly, PhysioNet is used for algorithm development rather than physical textile-fault validation. The WWBS Metrics dataset [27] is used separately to assess transfer to signals acquired from a textile-embedded garment. Body-worn IMU research has also shown that accelerometer data can support biomechanical analysis under controlled postures [28], which motivates inclusion of ACC as a motion-related modality but does not provide textile-degradation labels.

The following preprocessing pipeline was applied to the three raw signal modalities to suppress nonphysiological artifacts and establish a clean reference baseline for subsequent anomaly simulation.

Acceleration (ACC) Signal: The raw triaxial data were first combined into a vector magnitude to represent overall movement intensity:

$$M\;=\;\sqrt{\left\{x^{2}+\;y^{2}+\;z^{2}\right\}}$$

Subsequently, a robust Interquartile Range (IQR)-based filter was employed to remove spurious peaks caused by intense impacts, with the threshold set at

$$Q_{3}+3\times IQR$$

Finally, the signal was low-pass filtered at 10 Hz and down-sampled to 1 Hz to preserve the primary activity frequencies and reduce computational complexity.

Electrodermal Activity (EDA) Signal: A Butterworth filter bank was used to decompose the signal into the slowly varying Skin Conductance Level (SCL) component, representing tonic baseline drift, and the rapidly fluctuating Skin Conductance Response (SCR) component, representing phasic physiological arousal. To facilitate the subsequent simulation of baseline drift analogous to poor fabric–skin contact, a moving average method was applied to the SCL component for baseline correction, thereby highlighting genuine physiological fluctuations.

Heart Rate (HR) Signal: The raw PPG (photoplethysmogram)-derived signal is susceptible to motion artifacts. Therefore, Dynamic Time Warping (DTW) was employed to temporally align the HR and ACC signals, correcting for timing misalignments caused by device displacement or transmission latency. The processed three-channel signals (ACC magnitude, corrected EDA, and aligned HR) collectively formed the input features for the model and provided the individualized statistical basis (e.g., heart rate variability, exercise intensity distribution) for the subsequent generation of physiological anomalies.

### 2.2. Textile-Specific Anomaly Injection Strategy

**Figure 2:**
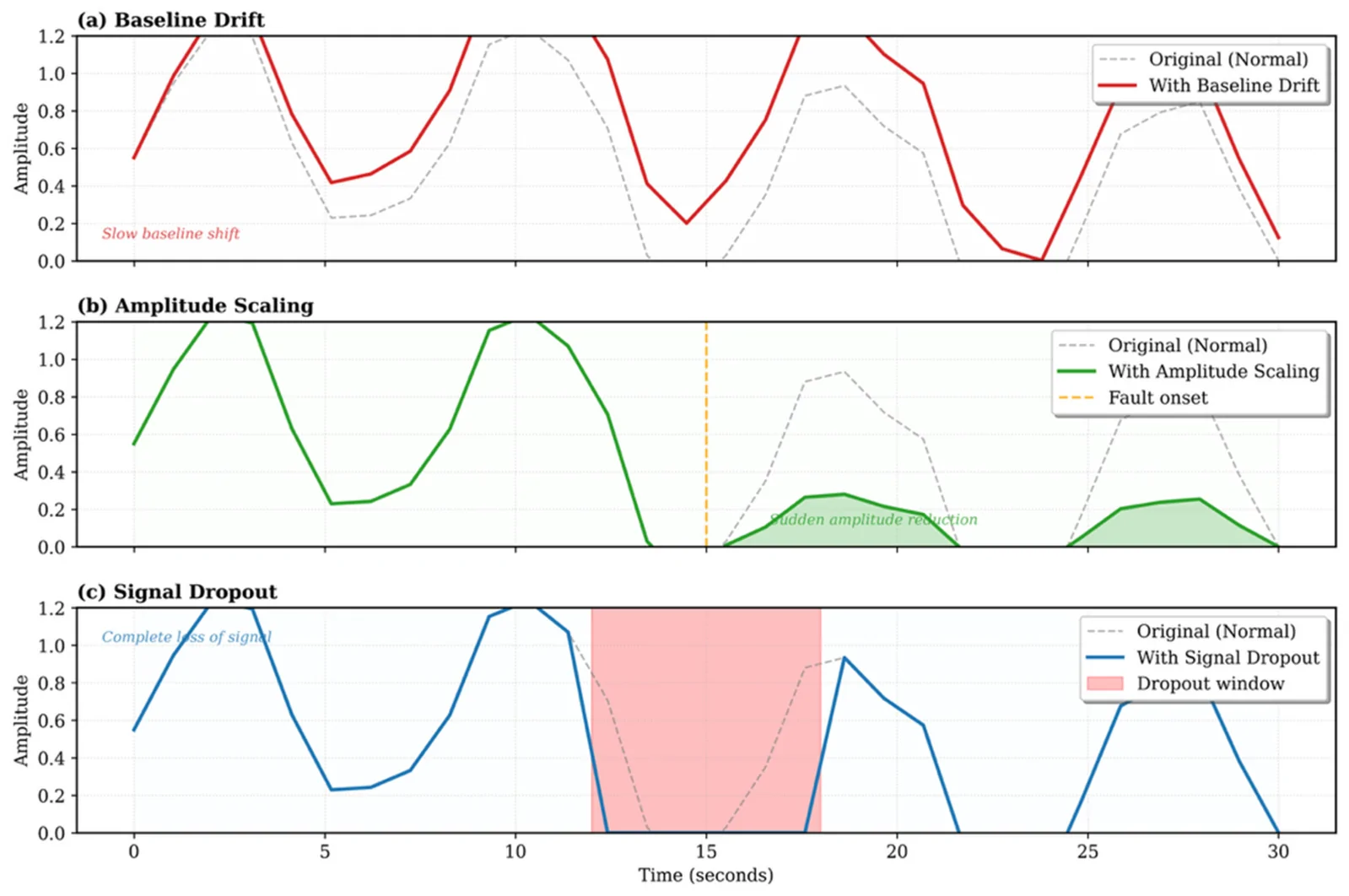
Representative textile-specific fault injection patterns: (**a**) baseline drift; (**b**) amplitude scaling; and (**c**) signal dropout.

A probabilistic window-level injection protocol was used to construct synthetic anomaly labels. Each window was assigned to the faulty class with probability 0.30. This rate was deliberately selected for supervised learning: a much lower event prevalence would create a strongly imbalanced training set and provide too few anomalous windows for learning the target fault patterns. Within faulty windows, one, two, or three modalities were affected with probabilities of 0.60, 0.30, and 0.10, respectively. This non-uniform allocation represents a severity-oriented simulation in which localized single-sensor faults are more common than concurrent multi-sensor faults; it is not an estimate of field-failure frequencies. For each affected modality, baseline drift, amplitude scaling, or signal dropout was sampled according to the modality-specific rules summarized in Table 1 and illustrated in Figure 2.

The three injected fault categories were selected as literature-grounded signal representations rather than empirically calibrated reproductions of physical degradation. Changes in fabric–skin contact resistance can produce slow baseline variation; deformation or coating changes can alter signal gain; and conductive-path or connector interruption can produce missing segments [7,8,9,10,11,12,13,14,29,30,31]. Synchronized electromechanical measurements of fabric strain sensors have shown that sensitivity and deformation patterns vary with loading rate under large deformation, providing direct evidence that mechanical loading can change the amplitude response of textile sensors [32]. A recent review of flexible tactile sensing also identifies environmental adaptability and multi-modal integration as unresolved system-level challenges [33,34].

These publications support the qualitative physical rationale for the injected fault categories but do not validate the numerical amplitudes, durations, or occurrence probabilities used here. The 30% faulty-window prevalence was a class-balancing design choice that retained a majority of normal windows while supplying enough positive examples for supervised training. The 60%/30%/10% distribution over one-, two-, and three-modality faults was a severity-oriented heuristic: one-modality faults represent localized deformation or contact failures, whereas two- and three-modality faults represent progressively broader disruption of sensing pathways. This controlled-fault approach is conceptually consistent with multi-modal fault-tolerance studies that inject faults to evaluate fusion under sensor degradation [24]. Neither study establishes these proportions as real-world textile-fault frequencies. The injection ranges therefore remain benchmark parameters rather than estimates of physical degradation distributions; sensitivity analyses across fault prevalences and physical calibration remain as future work.

After fault injection, each signal window is assigned a binary label. Fault-free windows are labeled as normal (y = 0), whereas windows containing at least one injected anomaly—baseline drift, amplitude scaling, or signal dropout—are labeled as anomalous (y = 1). These labels are used for supervised training and evaluation. The specific treatments for the three anomaly types are as follows:

Fault Type A: Baseline Drift: Primarily introduced in Heart Rate (HR) and Electrodermal Activity (EDA) signals by superimposing low-frequency trends along the temporal dimension (constituting 40% of synthetic anomalies), simulating slow signal shifts caused by changes in fabric–skin contact resistance or sensor slippage.

Fault Type B: Amplitude Scaling: Applicable to acceleration (ACC) and electrodermal activity (EDA) signals, implemented by multiplying the entire signal window by a randomly generated coefficient within the range of 0.5 to 1.5 (constituting 30%), simulating overall signal gain/attenuation due to fabric stretching or variations in sensing unit sensitivity.

Fault Type C: Signal Dropout: Can occur in all modalities, implemented by randomly selecting a 1 to 3 s interval within the signal window and setting its amplitude to zero (constituting 30%), simulating complete signal loss caused by instantaneous interruption of conductive paths or wireless transmission failure.

The injection protocol generates predefined combinations of baseline drift, amplitude scaling, and signal dropout for supervised model development. Because these patterns were not calibrated against signals collected during physical degradation tests, the resulting windows represent controlled test conditions rather than a comprehensive sample of faults encountered during use.

**Figure 3:**
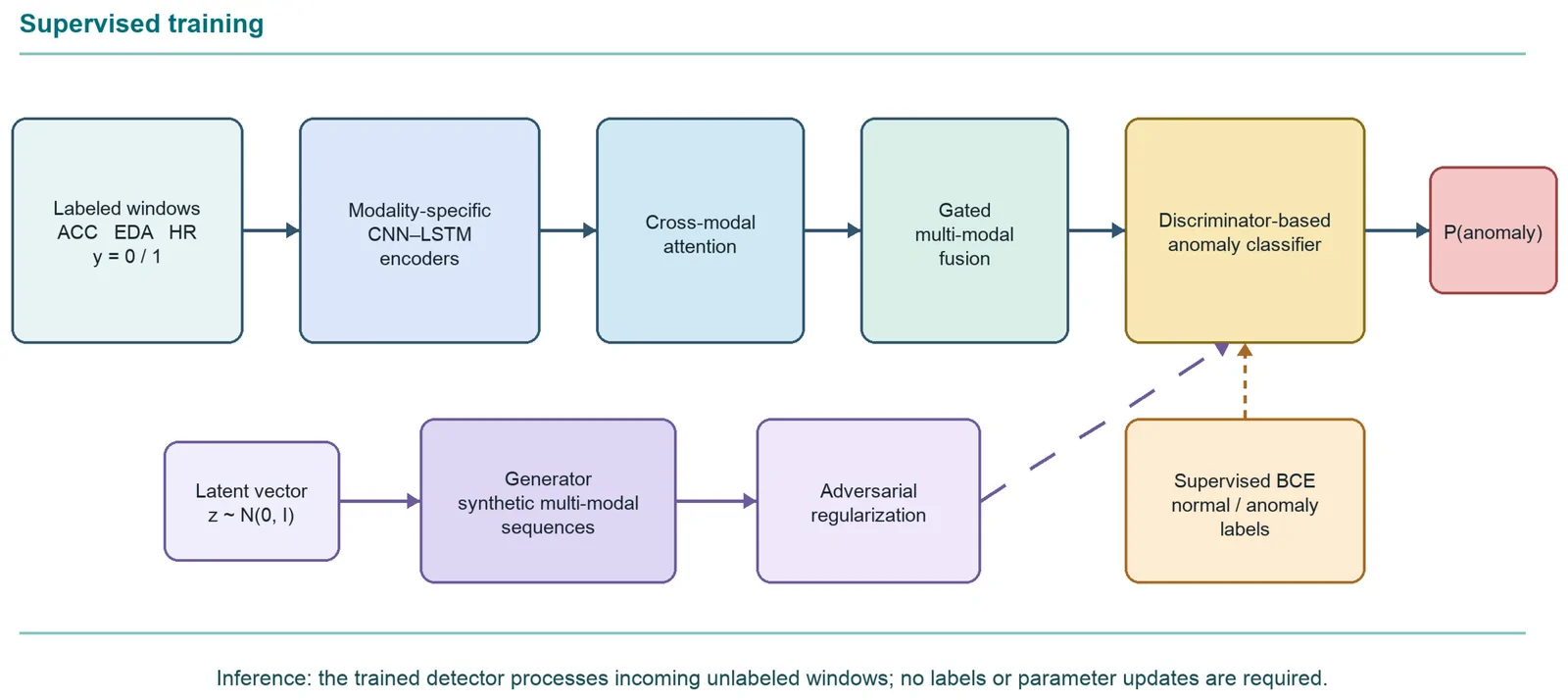
Architecture and training workflow of the proposed supervised multi-modal GAN-based anomaly detection framework. Modality-specific CNN-LSTM encoders extract temporal features from ACC, EDA, and HR; cross-modal attention and gated fusion form the multi-modal representation used by the Discriminator-based anomaly classifier. Normal/anomalous labels are used during training, whereas inference operates on unlabeled incoming windows.

To detect the injected anomaly patterns, this study constructs a supervised multi-modal framework based on a Generative Adversarial Network (GAN), as shown in Figure 3. The Generator learns to synthesize plausible multi-modal temporal sequences, while the convolutional-LSTM Discriminator provides the representation used for binary normal/anomaly classification. During training, normal and injected-anomaly windows are supplied with their binary labels, and adversarial learning regularizes the temporal representation learned by the classifier. The CNN layers capture local signal distortions, the LSTM models sustain temporal dependencies, and cross-modal attention integrates complementary ACC, EDA, and HR information. During inference, the trained detector assigns anomaly probabilities to incoming unlabeled windows; no labels or parameter updates are required.

The Generator adopts a sequence-to-sequence encoder–decoder architecture designed to synthesize multi-modal signal segments with realistic temporal dynamics from latent space noise. Its detailed structure is as follows:

1. Input Layer: Accepts a 100-dimensional noise vector **z** sampled from a standard normal distribution N(0,1).
2. Fully Connected Layer: Maps **z** to a 128-dimensional feature space, followed by Batch Normalization and a LeakyReLU activation function with a slope parameter of 0.2:
  $$h_{0}=\mathrm{LeakyReLU}\left(\mathrm{BatchNorm}\left(W_{0}z+b_{0}\right)\right)$$
3. Temporal Expansion Layer: Expands the feature vector **h**_0_ into a temporal sequence **H**_1_ ∈ R*^T^*^×128^ of length *T* = 30 via the RepeatVector operation, providing a foundation for subsequent temporal modeling.
4. LSTM Layer: Processes **H**_1_ using an LSTM layer with 128 units, outputting the full sequence **H**_2_ ∈ R*^T^*^×128^ to capture temporal dependencies:
  $$H_{2}={\mathrm{LSTM}}_{128}\left(H_{1}\right)$$
5. Convolutional Reconstruction Block:
  First Convolutional Layer: Uses 64 filters, a kernel size of 5, a stride of 1, and ’same’ padding, followed by Batch Normalization and LeakyReLU(0.2):
  $$H_{3}=\mathrm{LeakyReLU}\left(\mathrm{BatchNorm}\left({\mathrm{Conv}1D}_{64,5,1}\left(H_{2}\right)\right)\right)$$
  Second Convolutional Layer: Uses 32 filters, a kernel size of 5, a stride of 1, and ‘same’ padding, followed by Batch Normalization and LeakyReLU(0.2):
  $$H_{4}=\mathrm{LeakyReLU}\left(\mathrm{BatchNorm}\left({\mathrm{Conv}1D}_{32,5,1}\left(H_{3}\right)\right)\right)$$
6. Output Layer: A 1D convolutional layer with a kernel size of 5, stride 1, and ‘same’ padding maps the features to *C* channels (*C* = 3), with Tanh activation:
  $$X_{\mathrm{syn}}=\mathrm{Tanh}\left({\mathrm{Conv}1D}_{C,5,1}\left(H_{4}\right)\right)$$

The Generator is trained using the Adam optimizer (learning rate 2 × 10^−4^, β1 = 0.5) with an adversarial binary cross-entropy objective that encourages the generated multi-modal sequences to resemble the real signal distribution.

The Discriminator employs a hybrid architecture combining a Convolutional Neural Network with a Long Short-Term Memory network, designed to simultaneously capture local temporal distortions and violations of long-range dependencies indicative of sustained faults. Its design enables end-to-end anomaly probability assessment for both single-modal and multi-modal inputs. The detailed network structure is as follows:

1. Input Layer: Accepts temporal data of shape **X** ∈ R^*T*×*C*^, where *T* = 30 is the sequence length and *C* is the number of input modalities (*C* = 1 for single-modal experiments, *C* = 3 for multi-modal experiments).
2. Convolutional Feature Extraction Block:
  First Convolutional Layer: Uses 64 filters, a kernel size of 5, a stride of 2, and ‘same’ padding, followed by a LeakyReLU activation (slope 0.2) and a Dropout layer with a rate of 0.25:
  $$H_{1}={\mathrm{Dropout}}_{0.25}\left({\mathrm{LeakyReLU}}_{0.2}\left({\mathrm{Conv}1D}_{64,5,2}\left(X\right)\right)\right)$$
  Second Convolutional Layer: Uses 128 filters, a kernel size of 5, a stride of 2, and ‘same’ padding, followed by a LeakyReLU activation (slope 0.2) and a Dropout layer with a rate of 0.25:
  $$H_{2}={\mathrm{Dropout}}_{0.25}\left({\mathrm{LeakyReLU}}_{0.2}\left({\mathrm{Conv}1D}_{128,5,2}\left(H_{1}\right)\right)\right)$$
3. Temporal Dependency Modeling Layer: The extracted convolutional feature sequence **H**_2_ is fed into a 64-unit LSTM layer, which returns only the final hidden state **h**_lstm_ ∈ R^64^ of the sequence to model long-term dependencies:
  $$H_{\mathrm{LSTM}}={\mathrm{LSTM}}_{64}{\left(H_{2}\right)}_{\mathrm{last}}$$
  This is followed by a Dropout layer with a rate of 0.3 to prevent overfitting:
  $$h_{3}={\mathrm{Dropout}}_{0.3}\left(h_{\mathrm{lstm}}\right)$$
4. Output Layer: A fully connected layer with a Sigmoid activation function maps the features to a scalar probability *p* ∈ [0,1], indicating the predicted probability that the input window is anomalous:
  $$p=\sigma \left(W_{o}h_{3}+b_{o}\right)$$
  where *σ*(·) denotes the Sigmoid function.

The Discriminator is trained using the Adam optimizer (learning rate 2 × 10^−4^, β1 = 0.5). Its training objective combines adversarial discrimination with supervised binary cross-entropy based on the normal/anomalous labels. The resulting output is interpreted as the probability that an input window contains a signal anomaly.

To effectively leverage the intrinsic couplings between different physiological signals for enhanced identification of system faults, this study proposes an early fusion strategy based on cross-modal attention. This strategy first extracts deep temporal features of each modality via independent encoders, then explicitly models inter-modal interactions through an attention mechanism, and finally fuses them into a unified representation for the Discriminator. The detailed pipeline is as follows:

1. Modal Feature Encoding: The preprocessed trimodal signals (ACC, EDA, HR) are first mapped into high-dimensional temporal feature sequences via independent LSTM encoders:
  $$H_{\mathrm{ACC}}\in R^{T\times d},\;H_{\mathrm{EDA}}\in R^{T\times d},\;H_{\mathrm{HR}}\in R^{T\times d}$$
  where *d* is the feature dimension. Each feature sequence is then refined using layer normalization and a feedforward network:
  $$H_{m}=\mathrm{LayerNorm}\left(H_{m}+\mathrm{FFN}\left(H_{m}\right)\right),\;$$
  m∈{ACC,EDA,HR}
2. Cross-Modal Attention Modeling: To utilize known physiological couplings (e.g., movement–heart rate correlation), a cross-modal attention module is designed. Using ACC as the Query and HR as the Key and Value, the attention-weighted features are computed:
  $$Q=W_{q}H_{\mathrm{ACC}},\;K=W_{k}H_{\mathrm{HR}},\;V=W_{v}H_{\mathrm{HR}}$$
  $$\mathrm{Attention}\left(Q,K,V\right)=\mathrm{Softmax}\left(\frac{QK^{T}}{\sqrt{d_{k}}}\right)V$$
3. Gated Fusion Mechanism: A learnable gating unit is introduced to dynamically regulate the contribution of information from the cross-modal attention and the original primary modality:
  $$g=\sigma \left(W_{g}\left[H_{\mathrm{ACC}};\mathrm{Attention}\left(Q,K,V\right)\right]\right)$$
  $$F_{\mathrm{ACC}}=g⊙\mathrm{Attention}\left(Q,K,V\right)+\left(1-g\right)⊙H_{\mathrm{ACC}}$$
  where *σ* is the Sigmoid function and ⊙ denotes element-wise multiplication. Similarly, attention and fusion between other modality pairs can be constructed.
4. Multi-Head Self-Attention Fusion: The processed modal features **F**_ACC_, **F**_EDA_, **F**_HR_ are concatenated and fed into a multi-head self-attention module for final fusion, capturing global cross-modal dependencies:
  $$F_{\mathrm{final}}=\mathrm{MultiHeadAttention}\left(F_{\mathrm{ACC}},F_{\mathrm{EDA}},F_{\mathrm{HR}}\right)$$

The number of attention heads is set to 8, and a Dropout with a rate of 0.25 is applied to prevent overfitting. The final feature Ffinal is then passed to the Discriminator for the supervised binary anomaly classification decision.

The gated fusion module assigns data-dependent weights to the cross-modal attention output and the original modality features. Its contribution is evaluated by comparison with the attention-removed architectural variant reported in Table 2.

### 2.3. Temporal Dynamics and Oversmoothing Mitigation

A potential concern when applying cross-modal attention to signals with different intrinsic dynamics—such as fast-varying ACC and slow-varying HR—is temporal aliasing or oversmoothing. In the present framework, ACC and HR are temporally aligned via Dynamic Time Warping during preprocessing, and all modalities are resampled to a common 1 Hz rate. The LSTM encoders preserve sequential order without temporal pooling before the attention module; the full temporal resolution (30 time steps) is retained. The gated fusion mechanism is designed to reduce reliance on the attention output when modality dynamics diverge. In the component comparison reported in Table 2, the full model achieved higher F1-score, AUC, and accuracy than the attention-removed variant. These aggregate metrics do not directly test temporal aliasing or oversmoothing; dedicated signal-level analyses remain necessary.

To evaluate the framework, we compared the proposed model with conventional baselines, a reproduction of a recent generative wearable-data method, single-modality variants, and two controlled architectural variants. All data were partitioned into training (70%), validation (10%), and test (20%) sets following a subject-independent principle. The model was trained using the Adam optimizer (learning rate 1 × 10^−4^, β1 = 0.5) with a batch size of 64 for 100 epochs. Experiments were conducted on a workstation with an NVIDIA GeForce RTX 5090 GPU using the PyTorch (2.7) framework. The validation set was used for model selection only. For all reported evaluations, the Discriminator Sigmoid output was converted to a binary label using a pre-specified fixed threshold of 0.5; this threshold was not tuned on either the validation or test set. Once trained, the fixed model processes unlabeled test windows without further parameter updates.

## 3. Results and Discussion

### 3.1. Performance on the Primary Dataset (PhysioNet)

The proposed framework was compared with four conventional baseline detectors and with an independently reproduced implementation of the recent GenAI-based digital-twin method of Kamruzzaman et al. [23]. The latter was selected because it combines generative modeling with wearable-data anomaly detection. As the authors’ source code was not publicly available, the comparison implementation followed the methodological description in the publication; its numerical results may therefore differ from those of the original study. As shown in Table 3, the proposed model achieved an F1-score of 0.9197, compared with 0.7476 for the reproduced GenAI-based digital-twin method, 0.7692 for Decision Tree, 0.7280 for Isolation Forest, 0.6352 for Random Forest, and 0.6430 for the LSTM classifier. Repeated-run results and the paired comparison with the best single-modality model are reported in Table 4.

To quantify the contribution of combining sensor modalities, the full multi-modal model was compared with ACC-only, EDA-only, and HR-only variants (Table 5). The multi-modal model achieved an F1-score of 0.9197, compared with 0.6791 for the best-performing single-modality variant (ACC-only). This corresponds to an absolute increase of 0.2406 and a relative increase of 35.4%. The multi-modal model also achieved the highest accuracy among the variants reported in Table 5.

Among the single-modality variants, the ACC-only model achieved the highest accuracy (0.8847). The EDA-only model achieved a precision of 0.8405 and a recall of 0.5501, corresponding to a false-negative rate of 0.4499 on the present test set. The HR-only model achieved an F1-score of 0.6580. These aggregate modality-level metrics do not establish sensitivity to individual fault types because fault-type-stratified results were not calculated. On the present dataset, the multi-modal model achieved a higher F1-score, recall, and accuracy than each single-modality variant.

To quantify the contributions of the temporal encoder and cross-modal attention module, the full model was compared with two controlled architectural variants. In the first variant (attention removed), the cross-modal attention module was omitted and the modality-specific LSTM features were concatenated directly. In the second variant (LSTM replaced), the LSTM encoders were replaced with fully connected layers while the attention module was retained. Table 2 reports the resulting performance.

**Figure 4:**
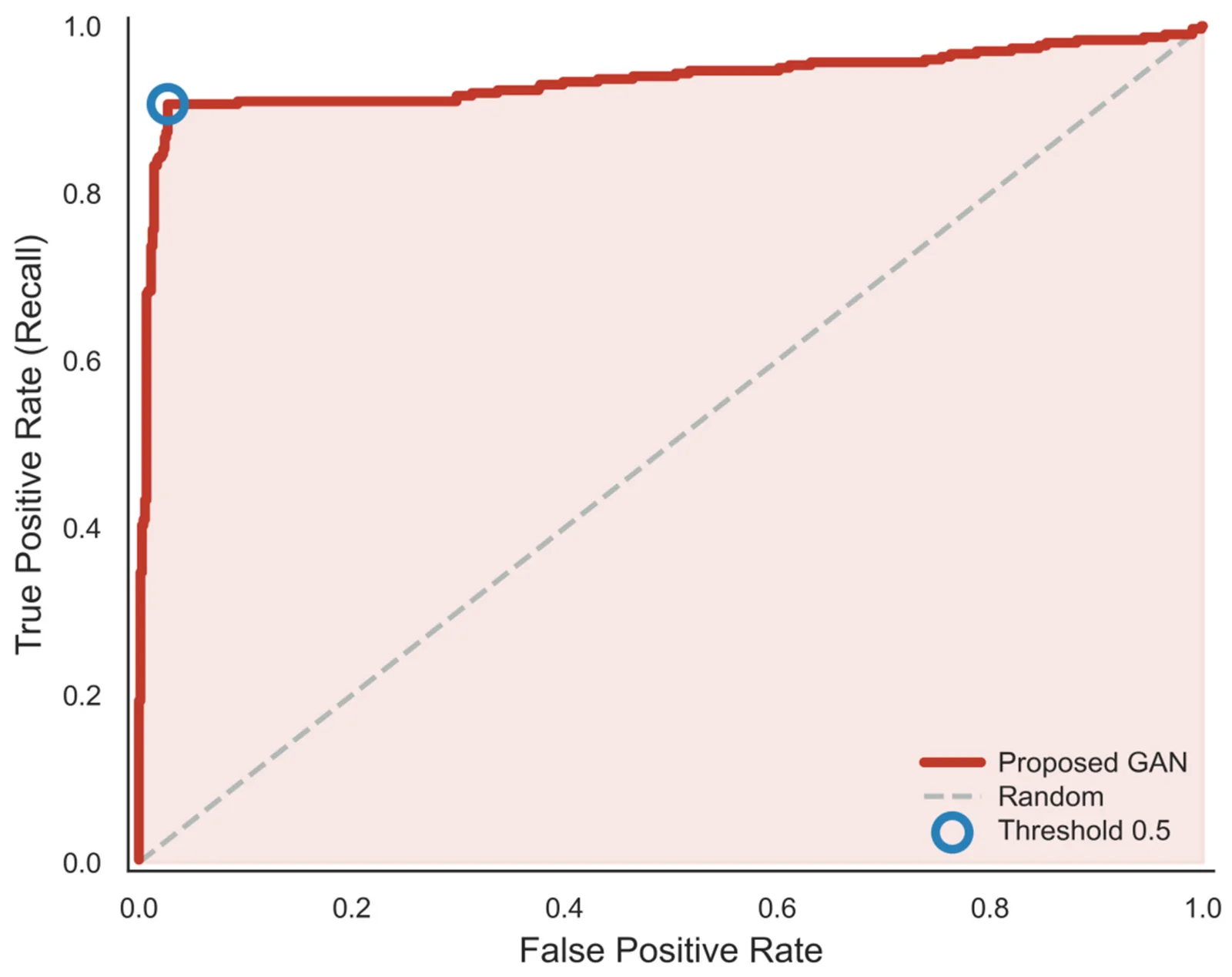
Receiver operating characteristic (ROC) curve of the proposed GAN on the PhysioNet test set (AUC = 0.9678). Anomalous windows are treated as the positive class; the blue marker indicates the pre-specified decision threshold of 0.5.

Removing cross-modal attention reduced the F1-score from 0.9197 to 0.8807, the AUC from 0.9678 to 0.8007, and the accuracy from 0.9347 to 0.8921; the relative F1-score reduction was 4.2%. The ROC curve of the full proposed model is shown in Figure 4. Replacing the LSTM encoders with fully connected layers reduced the F1-score to 0.8281 and the AUC to 0.7310, while accuracy was 0.8967; the relative F1-score reduction was 9.9%. For the statistical comparison, the full model and the best single-modality model (ACC-only) were evaluated with five random seeds (42–46), while the subject-independent data split was held fixed. On PhysioNet, the full model achieved a mean F1-score of 0.9143 (SD = 0.021), compared with 0.6775 (SD = 0.011) for ACC-only; the paired comparison was significant (*p* < 0.05). On WWBS, the full model achieved a mean F1-score of 0.8692 (SD = 0.017), compared with 0.6963 (SD = 0.013) for ACC-only; the paired comparison was significant (*p* < 0.05). Table 4 summarizes these repeated-run results.

### 3.2. Zero-Shot Cross-Dataset Evaluation on a Textile-Integrated Garment (WWBS Metrics)

To evaluate cross-dataset applicability to textile-integrated sensing hardware, we conducted zero-shot evaluation using the publicly available WWBS Metrics dataset [27]. Unlike the primary PhysioNet dataset, WWBS signals were acquired using a textile-embedded smart garment. The PhysioNet-trained model weights were applied without fine-tuning or parameter updates on WWBS.

Dataset adaptation. The WWBS Metrics dataset was collected using a Smartex smart garment with conductive fabric electrodes and embedded accelerometers. It provides ECG (250 Hz) and 3-axis ACC (25 Hz), but no native EDA or HR. The dataset characteristics are summarized in Table 6. HR was derived from ECG using Pan-Tompkins R-peak detection and interpolated to 1 Hz. For zero-shot evaluation, the two-modal ACC–HR configuration used the corresponding PhysioNet-trained weights, which remained fixed throughout WWBS evaluation. The same three anomaly types were injected into the WWBS ACC and derived HR signals to construct labeled evaluation windows. WWBS labels were used only to compute performance metrics and were not used for model training, model selection, threshold tuning, or fine-tuning. Thus, this experiment evaluates cross-dataset transfer to signals acquired from textile-integrated hardware rather than adaptation to WWBS. Table 6 summarizes the dataset characteristics.

Zero-shot results on textile-garment data. Table 7 reports the performance obtained by applying the PhysioNet-trained ACC–HR model directly to WWBS without fine-tuning, together with the corresponding single-modal results and the independently reproduced GenAI-based digital-twin baseline [23].

The zero-shot two-modal configuration achieved an F1-score of 0.8723 on WWBS when the PhysioNet-trained weights were applied without fine-tuning. Under the same WWBS protocol, the independently reproduced GenAI-based digital-twin baseline achieved an F1-score of 0.7277 (precision: 0.6539; recall: 0.7933).

Compared with the ACC-only result (F1-score = 0.6945), the two-modal ACC–HR configuration produced a 25.6% relative increase in F1-score. This result is limited to injected anomalies in ACC and ECG-derived HR. Differences from the PhysioNet result may arise from sensing hardware, participants, signal quality, modality availability, or data distribution, but the present design cannot isolate their individual effects. Because WWBS did not contain physically induced textile degradation or native EDA, this experiment evaluates two-modal cross-dataset transfer under controlled synthetic faults; it does not validate naturally occurring textile faults or the full ACC–EDA–HR framework.

#### 3.2.1. Limitations of Textile-Hardware Transfer Evaluation and Future Physical Validation

The WWBS Metrics dataset used for textile-hardware transfer evaluation is relatively small and lacks native EDA. Consequently, the WWBS results assess only the ACC–HR configuration and do not validate the full ACC–EDA–HR framework or textile-integrated EDA sensing. The two-modal model achieved an F1-score of 0.8723, but generalization requires larger datasets and physically induced faults. The present fixed-window architecture does not establish edge-deployment feasibility without measured parameter count, memory consumption, and inference latency. Future physical validation will collect synchronized ACC, EDA, and ECG/HR from a textile platform before, during, and after controlled stretching, repeated laundering, sweat and humidity exposure, cyclic fatigue, and induced connector or conductive-path interruptions. Electrical resistance and sensor outputs will be recorded to label degradation events, compare measured fault distributions with the synthetic patterns, recalibrate injection amplitudes, durations, and probabilities, and evaluate detection performance across repeated runs with confidence intervals. Cross-platform studies will include woven and knitted textiles and different electrode materials [29,30,31,35,36,37,38].

#### 3.2.2. Critical Discussion of Performance Degradation

The F1-score on WWBS (0.8723) was 0.0474 lower than that on PhysioNet (0.9197). Because the datasets differ simultaneously in sensing hardware, participants, signal quality, available modalities, and data distribution, the present experiment cannot attribute this difference to a specific factor. The WWBS result therefore describes two-modal transfer under the stated synthetic-fault protocol and requires confirmation using physically induced textile faults and additional smart-textile platforms.

#### 3.2.3. Future Work on Environmental Robustness and Long-Term Degradation

The current fault injection strategy does not include high-frequency electromagnetic interference (typical of industrial environments) or signal drift induced by sweat and humidity. Moreover, real-world smart textiles undergo progressive degradation after repeated washing and stretching cycles, and the current model does not automatically adapt to such gradual physical changes. Future work will therefore: (i) extend the fault model to cover environmental and industrial noise sources; (ii) investigate online fine-tuning and domain adaptation strategies to maintain detection accuracy over the product lifetime; and (iii) collect dedicated smart textile data under controlled textile stressors (laundering, stretching, sweat exposure) to validate these extensions.

#### 3.2.4. Computational Profile and Deployment Considerations

The proposed model contains approximately 0.8 million trainable parameters, and the final serialized weight file is approximately 4 MB. A single inference on the NVIDIA GeForce RTX 5090 workstation required approximately 0.8 ms. For a typical CPU-based computer, the corresponding latency is estimated to be approximately 8 ms; this is an engineering estimate, not a measured CPU benchmark. These measurements indicate a modest model footprint for workstation or computer-class edge deployment. They do not establish feasibility on a microcontroller, for which hardware-specific latency, peak memory, preprocessing cost, and energy consumption must be measured separately.

## 4. Conclusions

This study presented a supervised multi-modal GAN-based anomaly detection framework for signal reliability assessment in fabric-integrated smart textiles. Controlled injection of baseline drift, amplitude scaling, and signal dropout generated normal/anomalous labels for training and evaluation. By combining temporal encoding, cross-modal attention, and gated fusion, the framework distinguishes textile-mimicking signal degradation from physiologically plausible variation. Labels are required during training, whereas inference operates directly on incoming unlabeled signal windows.

On the subject-independent PhysioNet test set, the three-modal framework achieved a single-split F1-score of 0.9197, an absolute increase of 0.2406 over the best single-modality variant. Across five seeded runs, its mean F1-score was 0.9143 (SD = 0.021) on PhysioNet and 0.8692 (SD = 0.017) on WWBS; compared with ACC-only, both paired comparisons were significant (*p* < 0.05). The model contains approximately 0.8 million parameters and a 4 MB serialized weight file; single-window inference was approximately 0.8 ms on an NVIDIA GeForce RTX 5090. Applying the PhysioNet-trained ACC–HR model to WWBS without fine-tuning produced a single-split F1-score of 0.8723 under the same synthetic-fault protocol.

The primary limitation is that all anomaly labels, including those used for WWBS evaluation, were generated by controlled injection rather than by physically induced textile degradation. The present results therefore do not establish performance under laundering, stretching, sweat exposure, conductive-yarn fatigue, electrode delamination, or intermittent physical connections. The planned validation protocol will measure these stressors on textile-integrated ACC, EDA, and ECG/HR hardware, calibrate the synthetic fault model against the measured distributions, and then reassess model performance and edge-hardware feasibility.

## Figures and Tables

**Table 1:** Fault types, simulated signal behaviors, and corresponding textile-level causes.

Attribute	Baseline Drift	Amplitude Scaling	Signal Dropout
Simulated Signal Behavior	Low-frequency trend	Gain attenuation/amplification	Zero segments
Corresponding Textile-Level Cause	Fabric–skin contact resistance change due to sweating, yarn fatigue	Textile stretch, yarn slippage, coating degradation	Conductive path fracture, snap fastener failure, wireless interruption

**Table 2:** Performance of the full model and controlled architectural variants.

Model Variant	F1-Score	AUC	Accuracy
Ours	0.9197	0.9678	0.9347
Attention removed	0.8807	0.8007	0.8921
LSTM replaced	0.8281	0.7310	0.8967

**Table 3:** Performance comparison with conventional baselines and an independently reproduced recent generative wearable-data method.

Model	F1-Score	Precision	Recall
Ours	0.9197	0.9347	0.9052
Decision Tree	0.7692	0.7966	0.6048
Random Forest	0.6352	0.8734	0.4991
LSTM Classifier	0.6430	0.7676	0.5231
Isolation Forest	0.7280	0.8276	0.5863
GenAI-DT baseline (independent reproduction)	0.7476	0.7212	0.6231

Note: The GenAI-based digital-twin baseline was independently implemented from the published methodological description because source code was not available; its results may differ from those reported by Kamruzzaman et al. [23].

**Table 4:** Repeated-run F1-score results and statistical comparison with the best single-modality model.

Dataset	Model	Mean F1-Score	SD	Paired Comparison with ACC-Only
PhysioNet	Ours	0.9143	0.021	*p* < 0.05 (*n* = 5)
PhysioNet	ACC-only	0.6775	0.011	Reference
WWBS	Ours	0.8692	0.017	*p* < 0.05 (*n* = 5)
WWBS	ACC-only	0.6963	0.013	Reference

Note: Five runs used seeds 42, 43, 44, 45, and 46. For each seed, the subject-independent training/validation/test partition was fixed; only initialization and training stochasticity varied. Paired *t*-tests were conducted on the five matched F1-scores for Ours and ACC-only in each dataset (*p* < 0.05).

**Table 5:** Performance comparison between the full multi-modal model and single-modality variants.

Model	F1-Score	Precision	Recall	Accuracy
Ours	0.9197	0.9347	0.9052	0.9347
ACC-only GAN	0.6791	0.7723	0.6078	0.8847
EDA-only GAN	0.6656	0.8405	0.5501	0.7488
HR-only GAN	0.6580	0.8476	0.5366	0.8051

**Table 6:** Comparison of datasets: PhysioNet (model development) and WWBS Metrics (textile-hardware transfer evaluation).

Characteristic	PhysioNet (Primary)	WWBS Metrics (Validation)
Device type	Rigid wearable (Empatica E4)	Real textile-integrated garment (Smartex)
Sensor modalities	ACC, EDA, HR (3 modalities)	ACC, ECG (HR derived from ECG, 2 modalities)
Fault source	Synthetically injected (textile-mimicking)	Synthetically injected (textile-mimicking)
Physical textile degradation	No (simulated)	No (simulated)
Primary role	Model development and component-contribution analysis	Zero-shot cross-dataset evaluation on textile-integrated signals

**Table 7:** Zero-shot anomaly detection performance on WWBS Metrics using PhysioNet-trained weights without fine-tuning.

Model	F1-Score	Precision	Recall	Accuracy
Ours (ACC + HR, two-modal)	0.8723	0.8891	0.8562	0.8915
ACC-only (single-modal)	0.6945	0.7852	0.6231	0.8573
HR-only (single-modal)	0.6738	0.8124	0.5756	0.8169
GenAI-DT baseline (independent reproduction)	0.7277	0.6539	0.7933	—

Note: The GenAI-based digital-twin baseline was independently implemented from the published method because source code was unavailable. Accuracy was not calculated for this implementation.

## Data Availability

The PhysioNet Wearable Device Dataset is openly available at https://physionet.org/content/wearable-device-dataset/1.0.1/ (accessed on 11 August 2026). The WWBS Metrics dataset is openly available at https://doi.org/10.5281/zenodo.2671608. The analysis code used to reproduce the results of this study is available from the author, R.F. During the preparation of this manuscript/study, the authors used OpenAI Codex, GPT-5, for the purposes of English language refinement, improving manuscript organization and clarity, formatting assistance, and preparing a schematic figure based on the authors’ predefined model architecture. The authors have reviewed and edited the output and take full responsibility for the content of this publication.

## References

[1] Tat T., Chen G., Zhao X., Zhou Y., Xu J., Chen J. (2022). Smart textiles for healthcare and sustainability. ACS Nano.

[2] Massaroni C., Saccomandi P., Schena E. (2015). Medical smart textiles based on fiber optic technology: An overview. J. Funct. Biomater..

[3] Mohammadi R., Shirazi M., Moaref R., Jamalpour S., Tamsilian Y., Kiasat A. (2022). Protective smart textiles for sportswear. Protective Textiles from Natural Resources.

[4] Van Langenhove L. (2013). Smart textiles for protection: An overview. Smart Textiles for Protection.

[5] Zhang W., Luan S., Tian M., Qu L., Zhang X., Fan T., Miao J. (2025). Smart wearable fibers and textiles: Status and prospects. Nanoscale.

[6] Júnior H., Neves R., Monticeli F., Dall Agnol L. (2022). Smart fabric textiles: Recent advances and challenges. Textiles.

[7] Islam M., Hosen M., Islam T., Al Mamun M.A., Islam T. (2025). Advancements in functional smart and wearable textiles for sportswear applications. RSC Adv..

[8] Azani M., Hassanpour A. (2024). Electronic textiles (e-textiles): Types, fabrication methods, and recent strategies to overcome durability challenges (washability and flexibility). J. Mater. Sci. Mater. Electron..

[9] Huang J., Xie G., Xu X., Geng Z., Su Y. (2024). Degradable multilayer fabric sensor with wide detection range and high linearity. ACS Appl. Mater. Interfaces.

[10] Younes B. (2023). Smart e-textiles: A review of their aspects and applications. J. Ind. Text..

[11] Zaman S., Tao X., Cochrane C., Koncar V. (2018). Market readiness of smart textile structures—Reliability and washability. IOP Conference Series: Materials Science and Engineering.

[12] Zheng N., Wu Z., Lin M., Yang L.T., Pan G. (2009). Infrastructure and reliability analysis of electric networks for e-textiles. IEEE Trans. Syst. Man Cybern. Part C (Appl. Rev.).

[13] Mączka M., Pawłowski S., Plewako J. (2024). Modeling of the textronic structure using the Iterative Fundamental Solutions Method. Zeszyty Naukowe Politechniki Rzeszowskiej. Elektrotechnika.

[14] Ruckdashel R., Khadse N., Park J. (2022). Smart e-textiles: Overview of components and outlook. Sensors.

[15] Cai J., Deng W., Zhu Z., Zheng L., Sun M., Ma C.-B., Bai J., Bo X., Zhou M. (2025). A single carbon microyarn-based integrated wearable textile sweat sensor built into a hook-and-loop fastener. Anal. Chem..

[16] Ma Z., Bai R., Yu W., Li G., Chen H., Meng C. (2026). Flexible, breathable and cuttable zinc-air battery textile towards real wearable energy supply clothing. Compos. Part B Eng..

[17] Yildirim P., Birant D., Alpyildiz T. (2018). Data mining and machine learning in textile industry. Wiley Interdiscip. Rev. Data Min. Knowl. Discov..

[18] Nizam M., Khan A., Ullah A., Kawsari N. (2025). Self-learning smart textiles: AI-driven adaptive wearables for personalized health monitoring. Biomed. Mater. Devices.

[19] Duan S., Lin Y., Shi Q., Wei X., Zhu D., Hong J., Xiang S., Yuan W., Shen G., Wu J. (2024). Highly sensitive and mechanically stable MXene textile sensors for adaptive smart data glove embedded with near-sensor edge intelligence. Adv. Fiber Mater..

[20] Zhang X., Tang S., Ma R., Chen Z., Zhuo J., Cao L., Yang J., Yang G., Yi F. (2022). High-performance multimodal smart textile for artificial sensation and health monitoring. Nano Energy.

[21] Leal-Junior A., Avellar L., Frizera A., Marques C. (2020). Smart textiles for multimodal wearable sensing using highly stretchable multiplexed optical fiber system. Sci. Rep..

[22] Beeby S., Torah R., Wagih M., Isaia B., Black S., Saunders J., Yang K. (2025). Heterogeneous e-textiles: Materials, manufacturing and sustainability. Adv. Mater. Technol..

[23] Kamruzzaman M., Salinas J.S., Kolla H., Sale K.L., Balakrishnan U., Poorey K. (2025). GenAI-Based Digital Twins Aided Data Augmentation Increases Accuracy in Real-Time Cokurtosis-Based Anomaly Detection of Wearable Data. Sensors.

[24] Altinses D., Schwung A. (2025). Enhancing Fault Tolerance in Multimodal Learning: A VAE-Based Approach with Probabilistic Fusion. 2025 11th International Conference on Control, Decision and Information Technologies (CoDIT), Split, Croatia, 15–18 July 2025.

[25] Goldberger A., Amaral L., Glass L., Hausdorff J.M., Ivanov P.C., Mark R.G., Mietus J.E., Moody G.B., Peng C., Stanley H.E. (2000). PhysioBank, PhysioToolkit, and PhysioNet: Components of a new research resource for complex physiologic signals. Circulation.

[26] Hongn A., Bosch F., Prado L., Ferrández J.M., Bonomini M.P. (2025). Wearable physiological signals under acute stress and exercise conditions. Sci. Data.

[27] Orselli R. (2019). WWBS Metrics.

[28] Wang Z., Yuan X., Tang Y., Zhang S. (2026). Verification of the self-load assessment capability of inertial measurement units via lumbar musculoskeletal simulation. Smart Wearable Technol..

[29] Hosen M., Ferdous A., Ahmed S. (2024). Easily scalable and highly flexible machine knitted resistive pressure sensor for smart textile applications. J. Ind. Text..

[30] Grancarić A., Jerković I., Koncar V. (2017). Conductive polymers for smart textile applications. J. Ind. Text..

[31] Youssefi M., Motamedi F. (2019). An electrically conductive hybrid polyaniline/silver-coated polyester fabric for smart applications. J. Ind. Text..

[32] Yang B., Zhu X., Peng C., Zhou L., Wang F., Liu Z., Tang L., Jiang Z., Liu Y., Chen S. (2025). Synchronized measurement of electromechanical responses of fabric strain sensors under large deformation. Smart Wearable Technol..

[33] Ren H., Li W., Ding Y., Feng Y., Li H., Li J., Zhang J., Xiao H., Li J., Wang S. (2025). Flexible tactile sensors for enhancing robotic perception. Smart Wearable Technol..

[34] Cruz P., Achir N., Viana A. (2022). On the edge of the deployment: A survey on multi-access edge computing. ACM Comput. Surv..

[35] Kong X., Luo H., Huang G., Yang X. (2019). Industrial wearable system: The human-centric empowering technology in industry 4.0. J. Intell. Manuf..

[36] Patel S., Park H., Bonato P., Chan L., Rodgers M. (2012). A review of wearable sensors and systems with application in rehabilitation. J. Neuroeng. Rehabil..

[37] Ling Y., An T., Yap L., Zhu B., Gong S., Cheng W. (2020). Disruptive, soft, wearable sensors. Adv. Mater..

[38] Moon J., Ju B. (2024). Wearable sensors for healthcare of industrial workers: A scoping review. Electronics.

